# Platelet–Neutrophil interactions in *Klebsiella pneumoniae* invasive syndrome: The role of aspirin

**DOI:** 10.1080/21505594.2026.2634456

**Published:** 2026-02-19

**Authors:** Yin-Ting Lin, Chia-Chi Chang, Fang-Ju Chen, Chen-Hsiang Lee

**Affiliations:** a Division of Infectious Diseases, Department of Internal Medicine, Chiayi Chang Gung Memorial Hospital, Chiayi, Taiwan; b Microbiology Treatment and Research Center, Chiayi Chang Gung Memorial Hospital, Chiayi, Taiwan; c Division of Infectious Diseases, Department of Internal Medicine, Kaohsiung Chang Gung Memorial Hospital, Kaohsiung, Taiwan; d College of Medicine, Chang Gung University, Kaohsiung, Taiwan

**Keywords:** Platelet activation, platelet–neutrophil aggregates, hyperglycemia, thrombosis, antiplatelet therapy

## Abstract

*Klebsiella pneumoniae* invasive syndrome (KPIS), often arising from pyogenic liver abscesses, is characterized by metastatic infections and thrombotic complications. Diabetes mellitus (DM) is the most important risk factor for KPIS, as hyperglycemia promotes resistance of hypervirulent *K. pneumoniae* ;(hvKp) strains to phagocytosis and impairs neutrophil function. Given the interplay between platelet activation, inflammation, and thrombosis, aspirin, a well-established antiplatelet agent, has been associated with reduced incidence and recurrence of pyogenic liver abscesses in cohort studies. Platelets interact with neutrophils to form platelet – neutrophil aggregates (PNAs), which may contribute to KPIS pathogenesis. This study examined platelet – neutrophil interactions under hyperglycemic conditions using *in vitro* assays and *in vivo* models of diabetic mice infected with hvKp. High glucose concentrations significantly increased platelet activation, PNA formation, and bacterial survival. Salicylic acid, the bioactive metabolite of aspirin, reduced platelet activation and bacterial burden but did not impede PNA formation. Aspirin pre-treatment improved survival, reduced organ abscesses, and preserved tissue integrity in diabetic mice infected with hvKp. These results highlight the relationship between hyperglycemia, platelet activation, and immune dysregulation in KPIS, and support aspirin as a potential adjunctive therapy to mitigate thromboinflammatory complications of hvKp infection.

## Introduction

*Klebsiella pneumoniae* (Kp) has become a major cause of pyogenic liver abscess, especially in East Asia, and is increasingly reported worldwide, including in the United States [1,2]. These infections are often complicated by metastatic spread, such as meningitis, brain and lung abscesses, endophthalmitis, and necrotizing fasciitis. Together, these manifestations are referred to as *K. pneumoniae* invasive syndrome (KPIS) [3–5]. The development of KPIS depends on both bacterial and host factors. Among bacterial factors, hypervirulent *K. pneumoniae* (hvKp) strains, particularly serotypes K1 and K2, are strongly linked to KPIS [6,7]. On the host side, diabetes mellitus (DM) with poor glycemic control greatly increases the risk of severe and disseminated hvKp infection [8]. Hyperglycemia promotes capsule polysaccharide (CPS) gene expression, which makes hvKp more resistant to phagocytosis and increases the risk of invasive disease [9,10]. It also weakens neutrophil functions such as chemotaxis, phagocytosis, and bacterial killing, thereby increasing the risk of metastatic infection [11,12].

In addition to neutrophils, platelets are now recognized as important mediators of infection and inflammation [13]. Beyond their role in hemostasis, platelets interact with immune cells, regulate inflammatory signaling, and form platelet – leukocyte aggregates (PLAs). These aggregates contribute both to host defense and to thrombosis [14–16]. High levels of circulating PLAs are found in many pro-thrombotic conditions [17,18], and diabetic patients, especially those with vascular complications such as retinopathy, nephropathy, myocardial infarction, and stroke, show increased PLA levels [19]. Consistent with these observations, we previously found that patients with KPIS and poor glycemic control were more likely to develop hepatic venous thrombophlebitis [20], reflecting the frequent thrombotic complications of invasive Kp infection [21]. Because of this interplay between platelet activation, inflammation, and thrombosis in KPIS, platelet inhibition has been suggested as a therapeutic strategy. Retrospective studies have shown that aspirin, a widely used antiplatelet agent, can reduce the incidence and recurrence of pyogenic liver abscess [22,23], supporting its potential to limit both thrombotic and inflammatory responses in KPIS.

However, the links between platelet activation, PLA formation, and KPIS pathogenesis, as well as the role of aspirin, are not yet fully understood. This study aimed to clarify the contribution of platelet – leukocyte interactions to KPIS, especially under hyperglycemic conditions. Using *in vitro* assays and diabetic mouse models, we investigated how hvKp affects platelet activation and platelet – neutrophil aggregate (PNA) formation at different glucose levels, and we assessed the potential of aspirin to modify these effects.

## Materials and methods

### Ethics statement

This study was approved by the Institutional Review Board (IRB) of Chang Gung Memorial Hospital (IRB No. 201701769B0). Written informed consent was obtained from all participants in accordance with the Declaration of Helsinki.

### Bacterial strains

The *K. pneumoniae* KP-M1 strain (ST23, serotype K1) with a hypermucoviscosity phenotype was originally isolated from a patient with a liver abscess. An acapsular *K. pneumoniae* mutant, DT-X, was derived from DT-S (biotype edwardsii, serotype K1, ST23) through subculturing, which resulted in a non-hypermucoviscous phenotype. The absence of a capsule in DT-X was confirmed by India ink staining [24]. All strains were routinely cultured in Luria – Bertani (LB) medium at 37 °C. The hypermucoviscosity phenotype of the isolates was verified using a modified string test [24,25].

### Blood sample collection

Whole blood samples were collected from 10 healthy adult volunteers by venipuncture using a 22-gauge needle after light tourniquet application. For platelet activation and PNA analysis, blood was drawn into 3.2% sodium citrate tubes (Greiner Bio-One #454322, 2 mL); the first tube was discarded to minimize activation artifacts. Citrate was selected because it prevents premature platelet activation while preserving platelet responsiveness and leukocyte integrity. For neutrophil isolation, blood was drawn into heparinized tubes, which prevent coagulation and better preserve neutrophil function for subsequent assays. All samples were processed within 10 minutes of collection at room temperature with gentle handling.

### Platelet activation analysis

To evaluate platelet activation and reactivity, flow cytometry was used to measure CD61 and CD62P (P-selectin) expression [26]. Whole blood samples were stimulated under different conditions, including negative control (1× DPBS, Sigma-Aldrich #D1408), positive control with 20 μM ADP (adenosine 5′-diphosphate sodium salt, Sigma-Aldrich #A2754), bacterial stimulation (KP-M1 or DT-X, 10^8^ CFU/mL), and varying glucose concentrations (0%, 0.1%, and 0.5%) with or without 30 µg/mL salicylic acid (SA). For each condition, 0.5 mL of blood was mixed with 0.5 mL of stimulant solution for a final volume of 1.0 mL. Each group was incubated at 37 °C for 30 minutes. After stimulation, 10 μL of each sample was stained with anti-CD61/BB700 (BD #746050) and anti-CD62P/PE (BD #555524) for 15 minutes at room temperature in the dark. Samples were then fixed and red blood cells lysed with 0.5 mL of BD FACS Lysing Solution (BD #349202) at 4 °C for 30 minutes, centrifuged at 2000 × g for 5 minutes, washed with cold 1× DPBS, and resuspended in 0.5 mL of FACS buffer. Flow cytometry was performed on an LSR II Flow Cytometer (BD). Platelets were gated by forward- and side-scatter (FSC/SSC) characteristics and CD61 positivity, and CD62P expression was quantified within the CD61^+^ platelet population.

For soluble platelet activation markers, stimulated citrated whole blood was centrifuged at 1500 × g for 10 minutes to obtain platelet-poor plasma (PPP). The supernatant was then centrifuged at 10,000 × g for 10 minutes to yield platelet-free plasma (PFP). Plasma samples were aliquoted and stored at − 80 °C until analysis. P-selectin (Thermo Fisher Scientific #BMS219-4), platelet factor 4 (PF-4; Thermo Fisher Scientific #EHPF4), and β-thromboglobulin (β-TG/NAP-2; Thermo Fisher Scientific #EHPPBP) were quantified with commercial ELISA kits [27].

### Platelet-neutrophil aggregates analysis

PNA formation was assessed in citrated whole blood stimulated under the same conditions described above. After fixation and lysis of red blood cells with BD FACS Lysing Solution, samples were centrifuged, washed, and resuspended in FACS buffer, then stained with anti-CD42b/APC (BD #551061) to identify platelets and anti-CD11b/BV421 (BD #742637) plus anti-CD18/BV786 (BD #744555) to identify neutrophils [28]. After excluding debris and doublets, leukocytes were gated on FSC-A × SSC-A, and acquisition was stopped at 10,000 white blood cell (WBC) events. Platelets were displayed separately as CD42b^+^ events on CD42b × SSC-A for reference. PNAs were defined within the WBC gate as CD42b^+^ CD11b^+^ CD18^+^ triple-positive events. The percentage of PNAs was calculated as triple-positive events divided by the total WBC events acquired, with 10,000 cells used as the denominator for all samples. Data were analyzed with BD FACSDiva software (BD Biosciences, California, USA).

### Microscopic analysis of platelet aggregation and PNA formation

For microscopic analysis, PNA formation was evaluated by stimulating whole blood samples for 12 hours, preparing smears, staining with 10× diluted Giemsa solution for 30 minutes, and examining them under a Leica DM750 microscope at 400× and 1000× magnifications. Platelet aggregation was assessed using platelet-rich plasma (PRP) obtained by centrifuging stimulated whole blood at 180 × g for 15 minutes at room temperature (no brake). PRP smears were stained with 1× Giemsa solution for 30 minutes at room temperature and examined at 200×, 400×, and 1000× magnifications.

### Neutrophil bactericidal activity

To evaluate KP-M1 survival, purified neutrophils were incubated with KP-M1 (10^8^ CFU/mL; MOI 10) in 1× DPBS at 37 °C for 12 hours under four experimental conditions: KP-M1 alone, KP-M1 with 0.5% glucose, KP-M1 with 30 µg/mL SA, and KP-M1 with both 0.5% glucose and 30 µg/mL SA. After incubation, bacterial suspensions were serially diluted in sterile PBS and plated onto LB agar. Plates were incubated overnight at 37 °C, and colony-forming units (CFUs) were counted to quantify KP-M1 survival under each condition.

### In-vivo murine model

Male C57BL/6 (B6) mice (8 weeks old) were obtained from the National Laboratory Animal Center, Taiwan. Diabetes was induced by intraperitoneal injection of streptozotocin (STZ, 50 mg/kg), with hyperglycemia defined as fasting blood glucose above 350 mg/dL [29]. Starting at 11 weeks of age, mice in the treatment group received aspirin orally each day (0.5 mg/mL H_2_O, equivalent to 3–4 mg/kg/day), while control mice received sterile water. At 12 weeks, mice were infected with KP-M1 (10^9^ CFU/25 g mouse) by oral gavage. After infection, mice were monitored for 1 week to assess survival and infection-related outcomes, including liver abscess formation. Blood glucose levels were systematically monitored at predefined time points throughout the experimental period including before diabetes induction, prior to aspirin administration, before KP-M1 infection, and during the post-infection observation period to ensure sustained hyperglycemia in diabetic mice. At the end of the observation period, all mice were deeply anesthetized with Zoletil 50 (30 mg/kg) combined with Xylazine (5 mg/kg), administered intraperitoneally. The depth of anesthesia was verified by the absence of palpebral and pedal withdrawal reflexes before any terminal procedures. Under deep anesthesia, humane euthanasia was performed by a single, rapid cardiac puncture to ensure complete loss of consciousness and to minimize pain and distress, in accordance with the American Veterinary Medical Association Guidelines, and were approved by the Institutional Animal Care and Use Committee (IACUC) of Chang Gung Memorial Hospital (Approval No. 2,017,112,301). Tissue samples were subsequently collected for analysis.

Plasma samples were tested for P-selectin (CD62P) (Abcam #ab200014) using ELISA as a marker of platelet activation. Formalin-fixed tissues were processed for hematoxylin and eosin (H&E) staining to assess structural and inflammatory changes. All sections were independently reviewed by a board-certified pathologist blinded to the groups. A semi-quantitative scoring system was used (hepatocellular necrosis, inflammatory infiltration, and abscess formation, each graded 0–3), with total injury scores calculated as the sum of these parameters. Liver homogenates were also serially diluted in sterile PBS and plated on LB agar to determine bacterial burden, expressed as CFU per gram of tissue.

Mice were randomly assigned to experimental groups (B6 control, DM, ± KP-M1 infection, ± aspirin). Each group included 4–12 animals per experiment. The workflow of the animal experiments is shown in Figure S1. All experiments were carried out at the Chang Gung Memorial Hospital animal facility, in accordance with institutional and ARRIVE guidelines for the care and use of laboratory animals. A completed ARRIVE checklist is provided in the supplementary material.

### Statistical analyses

All data are presented as mean ± standard error of the mean (SEM). Comparisons between experimental and control groups were made using the Mann – Whitney U test. Kaplan – Meier survival analysis was used to compare survival, with statistical significance assessed by the log-rank test. GraphPad Prism (version 5.02, GraphPad Software, San Diego, CA, USA) was used for figures, and SPSS Statistics version 17.0 (SPSS Inc., Chicago, IL, USA) was used for all statistical analyses. A two-sided *p*-value < 0.05 was considered statistically significant.

## Results

### Platelet activation after KP-M1 incubation

**Figure 1.**
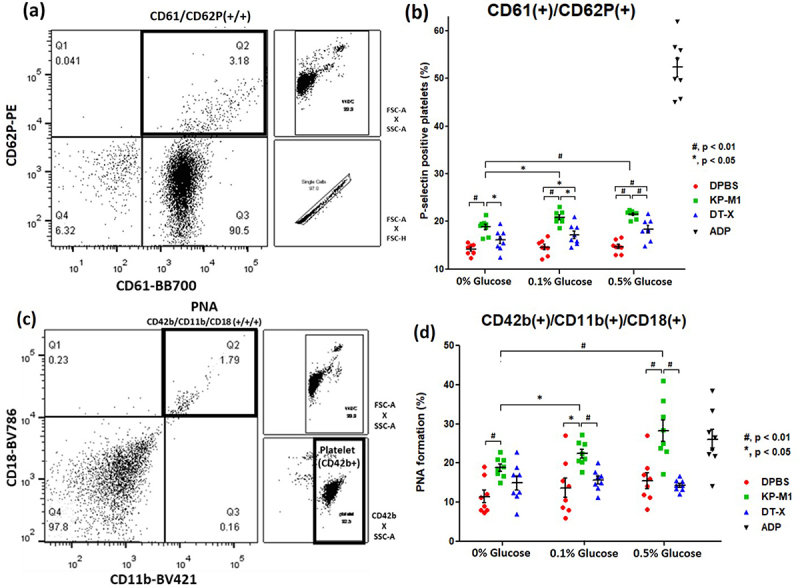
Platelet activation and platelet – neutrophil aggregate (PNA) analysis by flow cytometry. (a) Representative flow cytometry dot plot showing activated platelets (CD61^+^/CD62P^+^) in whole blood from healthy volunteers stimulated with DPBS (negative control). Samples (0.5 mL blood +0.5 mL stimulant) were incubated at 37 °C for 30 minutes and stained with anti-CD61 and anti-CD62P antibodies. Activated platelets were identified in the Q2 region as double-positive events. (b) quantitative analysis of CD61^+^/CD62P^+^ platelet populations under DPBS, 20 μM ADP (positive control), KP-M1 or DT-X (10^8^ CFU/mL), and glucose concentrations (0%, 0.1%, 0.5%). KP-M1 stimulation significantly increased platelet activation in a glucose-dependent manner (#, *p* < 0.01; *, *p* < 0.05, Mann – Whitney U test). Data represent eight independent experiments (*n* = 8).(c) Representative flow cytometry dot plots showing PNA formation (CD42b^+^/CD11b^+^/CD18^+^, Q2). Whole-blood samples were incubated under the same conditions and stained with anti-CD42b (APC), anti-CD11b (BV421), and anti-CD18 (BV786). After debris and doublet exclusion, leukocytes were gated on FSC-A×SSC-A, with acquisition stopped at 10,000 events. Platelets were displayed separately as CD42b^+^ events on CD42b×SSC-A for reference. PNAs were defined as CD42b^+^ CD11b^+^ CD18^+^ triple-positive events, expressed as the percentage of total WBC events (denominator: 10,000 cells per sample).(d) Quantitative analysis of PNA formation under different glucose concentrations. KP-M1 significantly enhanced PNA formation, with the highest levels observed at 0.5% glucose (#, *p* < 0.01; *, *p* < 0.05, Mann – Whitney U test). Data represent eight independent experiments (*n* = 8).

Flow cytometric analysis demonstrated that incubation with KP-M1 induced a marked increase in platelet activation, as reflected by a higher proportion of CD61^+^/CD62P^+^ platelets, compared with both the acapsular control strain DT-X and the DPBS control (Figure 1(a,b)). This effect was consistently observed under glucose-free conditions as well as at all tested glucose concentrations, indicating that KP-M1 alone is sufficient to trigger platelet activation.

Glucose supplementation further augmented KP-M1 induced platelet activation in a concentration dependent manner, with progressively increased CD61^+^/CD62P^+^ expression observed at 0.1% and 0.5% glucose. In contrast, glucose supplementation in the absence of KP-M1 (DPBS group) or following DT-X incubation did not significantly increase platelet activation, indicating that elevated glucose does not independently activate platelets but instead potentiates KP-M1-mediated activation. Statistical analysis confirmed significant differences in CD61^+^/CD62P^+^ populations between KP-M1–treated samples and DT-X or DPBS controls at all glucose concentrations.

**Figure 2.**
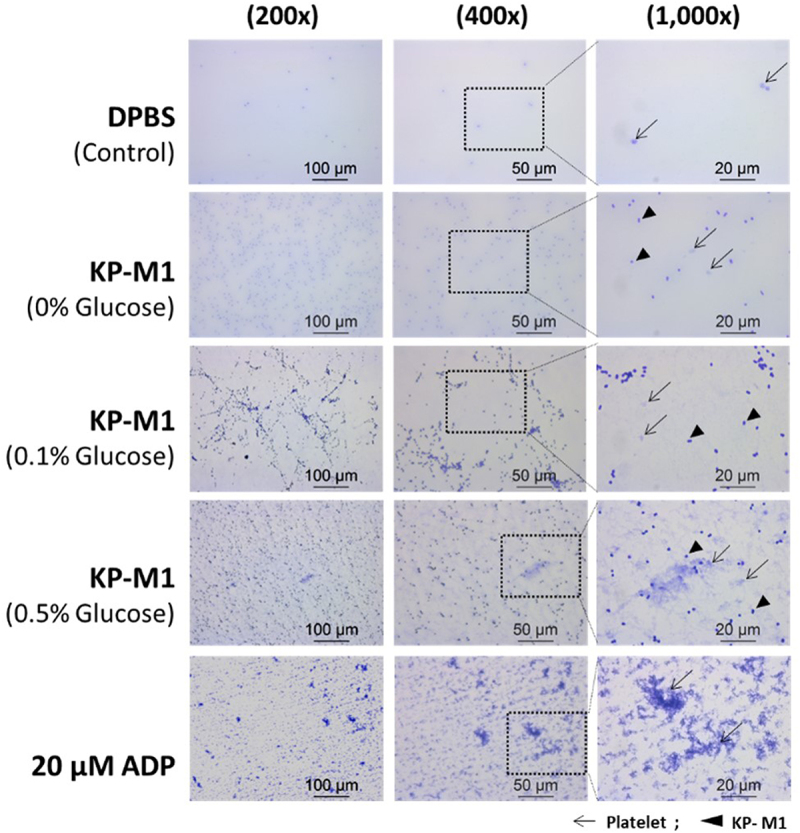
Microscopic observation of platelet aggregation in platelet-rich plasma (PRP) treated with KP-M1. DPBS-treated samples (control) showed minimal aggregation, while ADP-treated samples (positive control) exhibited extensive activation and clustering. KP-M1-treated samples showed platelet aggregation that increased with glucose concentration (0.1% and 0.5%). The most prominent clustering and filamentous structures were seen under 0.5% glucose.

Consistent with the flow cytometric findings, microscopic analysis of PRP samples revealed dense platelets aggregation and morphological features characteristic of activation following KP-M1 incubation, with these changes becoming more pronounced at higher glucose concentrations (Figure 2).

### PNA formation after KP-M1 incubation

In parallel with platelet activation, incubation with KP-M1 significantly promoted PNA formation, as demonstrated by an increased proportion of CD42b^+^/CD11b^+^/CD18^+^ events detected by flow cytometry (Figure 1(c,d)). This effect was specific to KP-M1, as neither DT-X nor DPBS controls induced comparable levels of PNA formation under any glucose condition tested.

**Figure 3.**
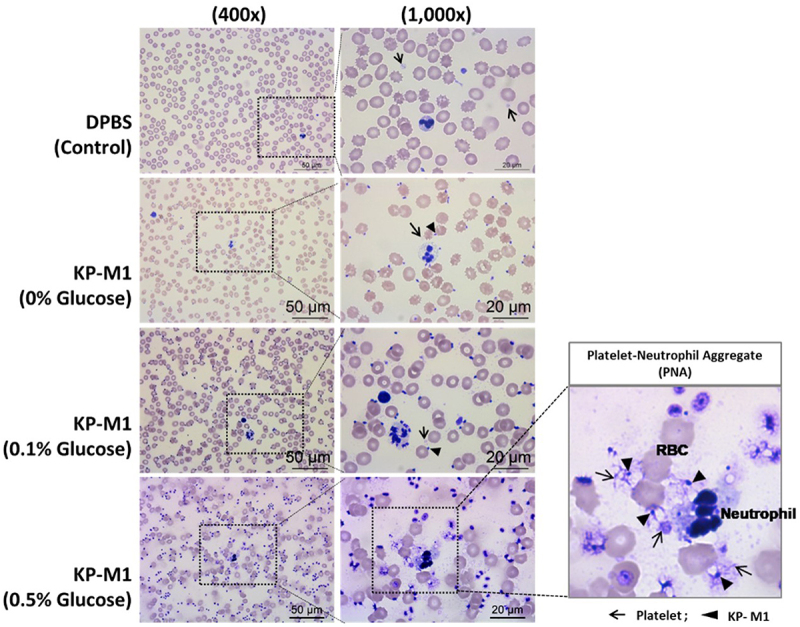
Microscopic examination of platelet – neutrophil aggregates (PNAs) in whole blood treated with KP-M1. Minimal aggregation was observed in the DPBS control. KP-M1-treated samples showed increased PNA formation in a glucose-dependent manner, with the most pronounced aggregation at 0.5% glucose. Dense platelet – neutrophil complexes were visible, as indicated by arrows and arrowheads.

Glucose supplementation further enhanced KP-M1–induced PNA formation in a concentration dependent manner, with the highest levels observed at 0.5% glucose. In contrast, baseline PNA formation in DPBS- and DT-X – treated samples remained relatively stable across glucose concentration, indicating that PNA formation requires KP-M1 exposure and that elevated glucose amplifies platelet – neutrophil interactions rather than acting as a primary trigger. Consistent with these findings, analysis of whole blood specimens revealed robust platelet – neutrophil interactions and aggregate formation following KP-M1 exposure, which were most pronounced under high-glucose conditions (Figure 3).

### Effect of salicylic acid on platelet activity and PNA formation

**Figure 4.**
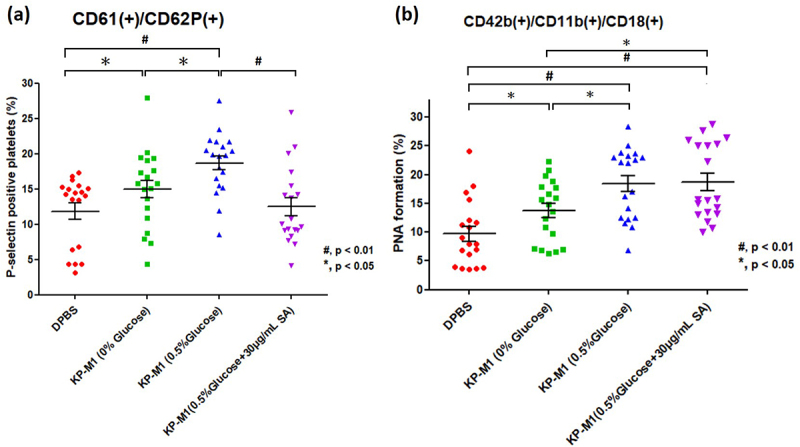
Effect of salicylic acid (SA) on KP-M1-induced platelet activation and PNA formation. (a) Flow cytometry analysis of platelet activation, shown as the percentage of CD61^+^/CD62P^+^ platelets under different stimulation conditions. KP-M1 in 0.5% glucose significantly increased platelet activation, which was markedly reduced by 30 μg/mL SA. (b) Quantitative analysis of PNA formation (CD42b^+^/CD11b^+^/CD18^+^) under different conditions. KP-M1 significantly increased PNA formation, particularly in 0.5% glucose. The addition of 30 μg/mL SA did not reduce PNA formation in 0.5% glucose despite its inhibitory effect on platelets. Data represent 19 independent experiments (*n* = 19). Statistical significance was assessed using the Mann – Whitney U test (#, *p* < 0.01; *, *p* < 0.05).

Salicylic acid (SA), the active component of aspirin, was tested for its effect on KP-M1–mediated platelet activation. Flow cytometry (Figure 4(a)) showed that adding 30 µg/mL SA significantly reduced CD61^+^/CD62P^+^ platelet populations under hyperglycemic (0.5% glucose) conditions. This reduction was also reflected in lower levels of activation markers such as P-selectin, PF-4, and β-TG (Figure S2). These results indicate that SA effectively reduces KP-M1–induced platelet hyperactivation in hyperglycemic conditions.

To examine whether this effect extended to PNA formation, we evaluated SA under the same hyperglycemic conditions. Despite lowering platelet activation, SA did not reduce PNA formation at 0.5% glucose (Figure 4(b)). This suggests that SA may influence neutrophils directly, promoting their aggregation with platelets or altering their functional activity.

We also tested the impact of SA on neutrophil bactericidal activity using CFU assays. As shown in Figure S3, KP-M1 survival increased markedly in 0.5% glucose. The addition of 30 µg/mL SA significantly lowered bacterial counts, suggesting enhanced neutrophil-mediated clearance of KP-M1. This finding is consistent with our earlier results [30].

### In-vivo murine model: aspirin in preventing KP-M1-induced invasive infections

**Figure 5.**
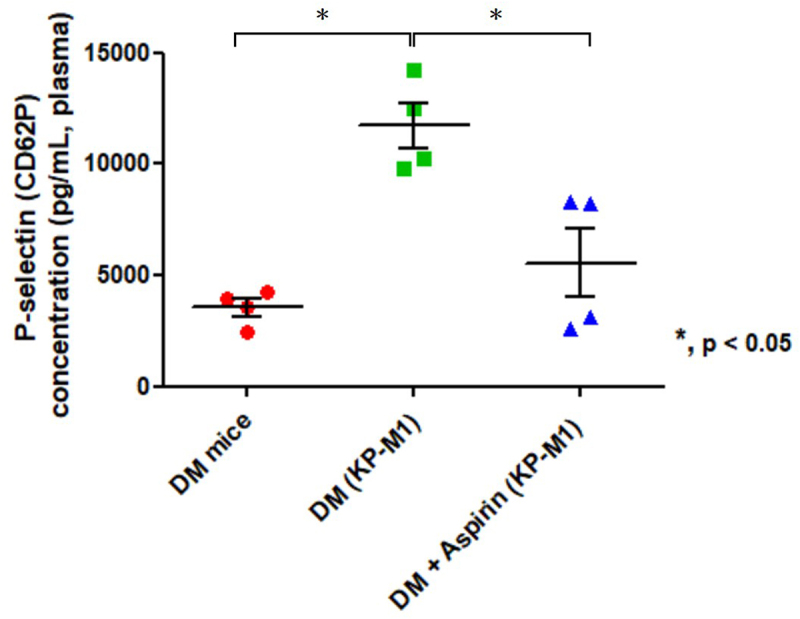
Plasma P-selectin (CD62P) levels in diabetic mice. Plasma was collected from uninfected diabetic (DM) mice, KP-M1–infected DM mice (DM + KP-M1), and KP-M1–infected DM mice pre-treated with aspirin (DM + aspirin + KP-M1). Plasma P-selectin levels were significantly higher in infected DM mice than in uninfected DM controls (*, *p* < 0.05, Mann – Whitney U test). Aspirin pre-treatment reduced P-selectin levels in infected DM mice, indicating attenuation of KP-M1–induced platelet activation. Data represent four mice per group (*n* = 4).

To evaluate the effect of aspirin on KP-M1–induced invasive infections, platelet activation was measured by plasma P-selectin (CD62P) levels in mice. P-selectin was markedly elevated in DM mice after KP-M1 infection (11,717 ± 1,031 pg/mL, mean ± SEM) compared with uninfected DM controls (3,551 ± 401 pg/mL, Figure 5). Aspirin pre-treatment reduced P-selectin concentrations in infected DM mice to 5560 ± 1,561 pg/mL, indicating its efficacy in attenuating platelet activation after KP-M1 infection.

**Figure 6.**
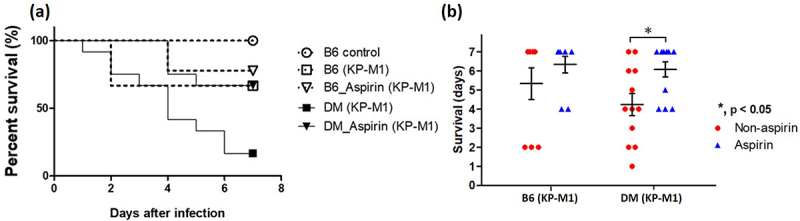
Effect of aspirin on survival in normoglycemic and diabetic mice after KP-M1 infection. (a) Kaplan – Meier survival curves of B6 and diabetic (DM) mice over 7 days after KP-M1 infection (1 × 10^9^ CFU/25 g). Uninfected control mice showed 100% survival, whereas KP-M1–infected mice had reduced survival. Aspirin pre-treatment (3–4 mg/kg) improved survival in both B6 and DM mice. (b) Dot plot of survival duration (days) in B6 and DM mice infected with KP-M1, with or without aspirin pre-treatment. Aspirin significantly prolonged survival in DM mice (*, *p* < 0.05, Mann – Whitney U test). Data represent 5 mice in the B6 control group, 9 in the B6 (KP-M1) group, 9 in the B6 + aspirin (KP-M1) group, 12 in the DM (KP-M1) group, and 12 in the DM + aspirin (KP-M1) group.

**Figure 7.**
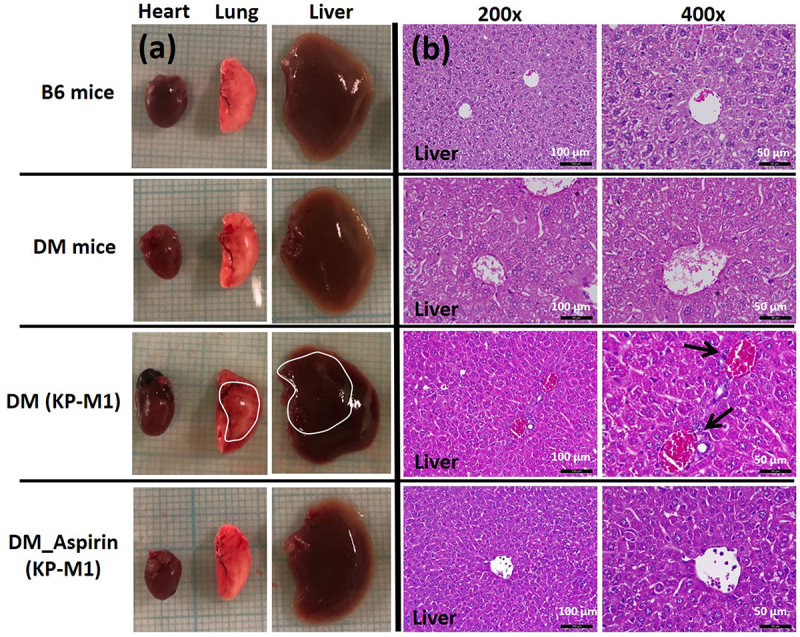
Postmortem and histological analysis of organ damage in mice. (a) Gross examination of heart, lung, and liver tissues from B6 mice, diabetic (DM) mice, DM mice infected with KP-M1, and DM mice pre-treated with aspirin before KP-M1 infection. Abscesses were visible in the lungs and liver of infected DM mice (outlined in white) but absent in aspirin-pretreated DM mice. (b) Representative H&E-stained liver sections at 200× and 400× magnification. KP-M1–infected DM mice showed disorganized hepatic cells and nuclear aggregation near endothelial gaps (black arrows). Aspirin-pretreated infected DM mice displayed preserved liver structure, similar to uninfected controls. Scale bars: 100 μm (200×) and 50 μm (400×). Blinded semi-quantitative scoring of liver injury (0–3 for necrosis, inflammatory infiltration, and abscess formation; total = sum of sub-scores) was performed by an independent pathologist. Scores were assessed in 5 mice per B6 and DM control group and in 12 mice per DM + KP-M1 or DM + aspirin (KP-M1) group. Results are shown as mean ± SEM. B6 and DM controls had minimal injury (0.8 ± 0.4 and 1.2 ± 0.5), KP-M1–infected DM mice had the highest scores (6.4 ± 0.7), and aspirin pre-treatment reduced liver injury (2.3 ± 0.5; *p* < 0.01 vs. DM + KP-M1, Mann – Whitney U test).

Survival was then assessed in normoglycemic (B6) and DM mice. Uninfected control mice had 100% survival during the observation period. In contrast, KP-M1 infection significantly reduced survival in both B6 and DM mice, with mean survival of 5.3 ± 0.8 days and 4.3 ± 0.6 days, respectively. Aspirin pre-treatment extended survival, with infected B6 and DM mice surviving 6.3 ± 0.4 days and 6.1 ± 0.4 days, respectively (Figure 6(a)); however, statistical significance was observed only in the DM group (Figure 6(b)). Collectively, these results indicate that aspirin attenuates the severity of KP-M1 infection *in vivo*, with a more pronounced protective effect under hyperglycemic conditions. Postmortem examination showed extensive abscesses in the liver and lungs of infected DM mice. In contrast, aspirin-treated DM mice did not develop visible abscesses, suggesting a protective effect against tissue damage (Figure 7(a)). Histopathology of the liver confirmed structural injury in infected DM mice, including disrupted hepatic cells and abnormal nuclear aggregation near endothelial gaps (Figure 7(b), black arrows). Aspirin-pretreated DM mice showed preserved liver architecture similar to that of uninfected controls. Semi-quantitative scoring by a blinded pathologist supported these findings: B6 and DM controls had minimal injury (0.8 ± 0.4 and 1.2 ± 0.5, respectively), infected DM mice had the highest injury scores (6.4 ± 0.7), and aspirin pre-treatment significantly reduced injury (2.3 ± 0.5; *p* < 0.01 vs. DM + KP-M1).

**Figure 8.**
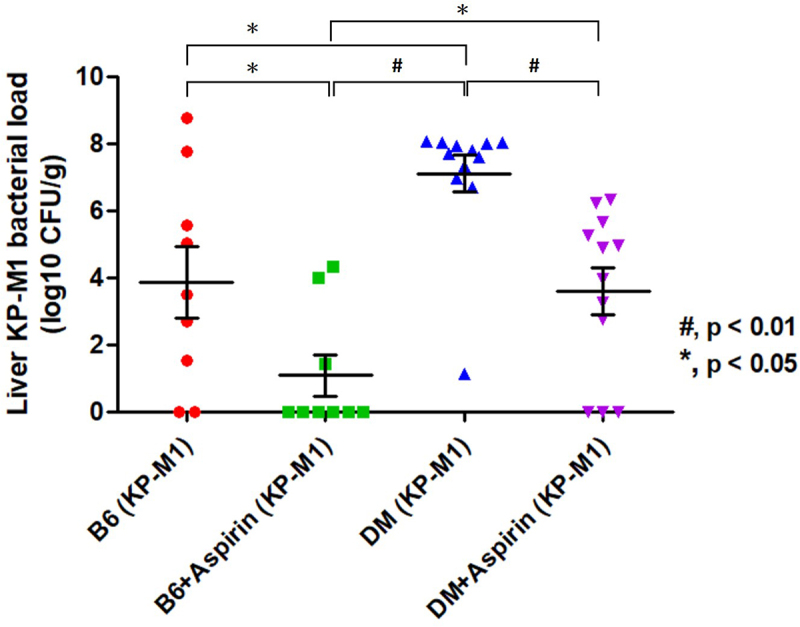
Aspirin reduces liver bacterial burden in KP-M1–infected mice. Dot plot of log-transformed CFU per gram of liver tissue in B6 and diabetic (DM) mice with or without aspirin pre-treatment. Infected DM mice without aspirin [DM (KP-M1)] had the highest bacterial burden. Aspirin pre-treatment reduced bacterial loads in both B6 and DM mice infected with KP-M1 (#, *p* < 0.01; *, *p* < 0.05, Mann – Whitney U test). Data represent 9 mice in the B6 (KP-M1) group, 9 in the B6 + aspirin (KP-M1) group, 12 in the DM (KP-M1) group, and 12 in the DM + aspirin (KP-M1) group.

To quantify bacterial burden in the liver, homogenates were prepared, plated, and expressed as CFU per gram of tissue. Infected DM mice without aspirin had the highest bacterial loads, while aspirin pre-treatment significantly reduced bacterial counts in both B6 and DM mice (Figure 8). Among all groups, aspirin-pretreated B6 mice showed the lowest bacterial burden, highlighting aspirin’s effectiveness in reducing KP-M1 infection and limiting liver damage.

## Discussion

In this study, we investigated platelet – neutrophil interactions in KPIS under hyperglycemic conditions and assessed the therapeutic potential of aspirin in modulating hvKp infection. A previous murine model has shown that DM creates a permissive environment that facilitates translocation of Kp strains from the intestines into the bloodstream [31]. Poor glycemic control has also been shown to stimulate CPS biosynthesis and increase cps gene expression in hvKp strains, leading to resistance to phagocytosis and progression of invasive disease [9,10]. Hyperglycemia further compromises immune function by impairing neutrophil chemotaxis, phagocytosis, and microbicidal activity, which increases the risk of vascular complications and infections in diabetic patients [11,12]. Consistent with these reports, our study demonstrated that hyperglycemia (0.5% glucose) significantly intensified hvKp-induced platelet activation, increased PNA formation, and promoted bacterial survival. These findings suggest that hyperglycemia exacerbates hvKp-driven immune dysregulation and contributes to KPIS.

Recent studies have highlighted the broader role of platelets beyond hemostasis, showing their involvement in inflammatory and infectious responses [13,16]. Platelets recognize pathogens through Toll-like receptors and release antimicrobial peptides [16,32]. They also enhance leukocyte activity through direct contact, cytokine release, and microvesicle production, facilitating phagocytosis and bacterial clearance [33,34]. Gautam et al. reported that platelets alone did not significantly inhibit Kp proliferation; however, thrombin-activated platelets enhanced monocyte-mediated killing via platelet – monocyte aggregation [35]. This process occurs when activated platelets adhere to monocytes through P-selectin and its ligand PSGL-1, thereby strengthening immune interactions and increasing bactericidal efficiency [36]. Similarly, Deppermann and Kubes reviewed the role of platelets in inflammation and infection, emphasizing their interaction with neutrophils to form PNAs [16]. These aggregates promote neutrophil adhesion to the endothelium, production of reactive oxygen species, and formation of neutrophil extracellular traps (NETs) [16,32,37]. While NETs capture and neutralize pathogens, excessive or prolonged NET formation may cause tissue injury and thrombosis [16,38]. Previous studies have shown that Kp infection with poor glycemic control is strongly associated with thrombotic complications, especially hepatic venous thrombophlebitis [20,21]. Elevated circulating PLA levels have also been linked to pro-thrombotic disorders [17,18], and diabetic patients with vascular complications show higher PLA levels [19]. Based on these findings, we propose that hvKp infection combined with hyperglycemia increases PLA formation, contributing to the heightened thrombotic risk observed in KPIS. In our study, we observed strong platelet activation and increased PNA formation under hyperglycemia after KP-M1 incubation. In line with this, our murine model showed organ abscess formation and liver injury. These results support prior evidence that platelet activation plays a dual role in infection, promoting host defense while also driving vascular complications [16,35].

Our previous work showed that SA treatment significantly reduced CPS production in hvKp, thereby enhancing leukocyte-mediated phagocytosis and bacterial clearance [30]. Retrospective studies have also reported that aspirin lowers both the prevalence and recurrence of Kp-PLA in diabetic patients [22,23]. Building on these findings, our *in vitro* and *in vivo* studies demonstrated that aspirin reduced platelet activation, decreased bacterial viability, and improved survival in a diabetic murine model of invasive Kp infection. These results suggest that modulating platelet activation and thrombosis may be a viable therapeutic strategy to limit the inflammatory and thrombotic complications of KPIS.

Although aspirin’s inhibitory effect on platelet activation is well established, its influence on PNA formation appears more complex, suggesting mechanisms beyond platelet suppression alone. Contrary to our initial expectation, SA did not reduce PNA formation under hyperglycemic conditions, despite lowering platelet activation markers. This finding indicates that aspirin may directly affect neutrophil activity within PNAs rather than acting only through platelets. For example, López-Farré et al. showed that aspirin not only inhibits platelet thromboxane A_2_ synthesis but also promotes neutrophil-mediated platelet inactivation via a nitric oxide (NO)/cGMP-dependent pathway [39]. This highlights the role of neutrophils in platelet regulation and adds complexity to PNA dynamics in KPIS. Additional evidence suggests that PNAs contribute to acute lung injury by promoting platelet sequestration and tissue damage [40]. Aspirin can mitigate such injury by inducing aspirin-triggered lipoxin, which reduces PNA formation and limits damage through the lipoxin A4 receptor pathway [40]. In contrast, our findings showed that SA did not decrease PNA formation, even though it reduced bacterial load and improved survival in diabetic mice. This raises questions about the role of PNA in KPIS, whether it primarily contributes to immune protection or instead worsens disease by driving thrombosis and inflammation. These results highlight the dual role of platelet – neutrophil interactions in immunity and pathology. Further studies are needed to clarify how aspirin modulates PNA function in hvKp infection and whether its overall effect is beneficial or harmful in KPIS.

In this study, we established an *in vitro* model that reproduced key aspects of hvKp infection under hyperglycemic conditions. This system allowed us to systematically examine how hvKp and high glucose affected platelet activation, PNA formation, and neutrophil bactericidal activity, as well as to test the effects of SA treatment. In addition to flow cytometric analysis, we provided direct microscopic visualization of platelet activation and PNA formation to qualitatively complement our findings. This experimental platform not only improves mechanistic understanding but also supports previous clinical observations in a reproducible *in vitro* framework. We also developed a diabetic murine model to assess the effect of aspirin pre-treatment in hvKp infection. Unlike traditional models that use intraperitoneal or localized inoculation, our approach applied oral gavage, which better simulates a clinically relevant infection pathway. In this model, hvKp infection significantly increased mortality in diabetic mice and caused widespread organ involvement. In addition to pulmonary and hepatic abscesses, severe infection spread to the periorbital region and cervical soft tissues, leading to edema and mass formation (Figure S4). Homogenization and bacterial plating confirmed a high KP-M1 burden in these infected tissues. Together, these results demonstrate the strength of our animal model in replicating key clinical features of KPIS and provide a valuable platform for studying disease progression and testing potential therapies.

This study has several limitations that should be considered. Although SA reduced platelet activation, it did not inhibit PNA formation under hyperglycemia, complicating its role in modulating platelet – neutrophil interactions in KPIS. This may reflect other immune regulatory mechanisms, but the exact pathways remain unclear. Still, our *in vitro* and *in vivo* experiments showed that SA and aspirin enhanced neutrophil activity, reduced bacterial burden, and improved survival in diabetic mice. Aspirin pre-treatment also lessened organ abscess formation and preserved liver architecture, supporting its promise as a therapeutic option for hvKp infection in diabetic hosts. Moreover, this work focused only on aspirin and did not evaluate other antiplatelet agents, such as clopidogrel, prasugrel, or ticagrelor. Whether these drugs exert similar or distinct effects on PNA dynamics and infection outcomes remains unknown. In addition, arachidonic acid stimulation, a standard assay for aspirin responsiveness, was not performed and should be included in future studies. Soluble P-selectin is not entirely platelet-specific, as it can also be released from activated endothelial cells; however, β-TG, PF4, and soluble P-selectin all showed consistent results, supporting our interpretation of enhanced platelet activation.

## Conclusion

This study demonstrates that hvKp markedly increases platelet activation and PNA formation, and that these effects are further amplified by hyperglycemia. Aspirin administration reduced platelet activation, lowered bacterial viability, and improved survival in diabetic mice infected with hvKp. These results highlight the close link between hyperglycemia, platelet activation, and immune dysregulation in KPIS, and support aspirin as a potential adjunctive therapy to reduce thromboinflammatory complications in hvKp infection.

## Data Availability

All datasets generated for this study are included in this article and the supplementary files. The data that support the findings of this study are available in figshare at https://doi.org/10.6084/m9.figshare.29094755.v4 [41].
